# Amino-Functionalized Carbon Nanodots Inhibit Biofilms and Infections in a Burn Wound Model, Both Caused by *Staphylococcus aureus*, *Escherichia coli*, *Candida albicans*, and *Aspergillus brasiliensis*

**DOI:** 10.1155/ijm/8824725

**Published:** 2025-10-14

**Authors:** Virginia Giusti, Alessandro Camilli, Alessio Valletta, Andrea Giammarino, Fabrizio Vetica, Elisa Sturabotti, Francesca Leonelli, Giovanna Simonetti

**Affiliations:** ^1^Department of Environmental Biology, Sapienza University of Rome, Rome, Italy; ^2^Department of Chemistry, Sapienza University of Rome, Rome, Italy; ^3^Department of Public Health and Infectious Diseases, Sapienza University of Rome, Rome, Italy; ^4^Centre for Cooperative Research in Biomaterials (CIC biomaGUNE), Donostia-San Sebastián, Spain

**Keywords:** *Aspergillus brasiliensis*, *Candida albicans*, carbon dots, *Escherichia coli*, *Staphylococcus aureus*, wound infections

## Abstract

Burn wounds are debilitating injuries that contribute significantly to global morbidity and mortality. The disruption of skin integrity elevates the risk of infection, which can impede healing and potentially lead to sepsis. Furthermore, antibiotic resistance, primarily driven by biofilm formation, poses a major challenge to effective treatment. This study is aimed at evaluating the potential of positively charged carbon dots (CDs) in inhibiting biofilm formation, with possible applications in wound care. Specifically, carbon nanodots (CDs-NH_2_) were tested in vitro against both planktonic cells and biofilms formed by a range of pathogens, including the Gram-positive bacterium *Staphylococcus aureus*, the Gram-negative bacterium *Escherichia coli*, the yeast *Candida albicans*, and the mold *Aspergillus brasiliensis*. Additionally, the activity of CDs-NH_2_ was assessed against polymicrobial biofilms composed of *S. aureus* and *C. albicans*. The in vivo efficacy of CDs-NH_2_ was evaluated using the *Galleria mellonella* burn wound infection model for both monomicrobial and polymicrobial infections. The ability of CDs-NH_2_ to penetrate fungal cells was demonstrated by fluorescence microscopy analysis. Biomass quantification showed that CDs-NH_2_ reduced biofilm formation by over 50% for *C. albicans*, *E. coli*, and *A. brasiliensis*, as well as for *C. albicans*–*S. aureus* cocultures, at concentrations below 62.5 *μ*g/mL. The in vivo studies further confirmed the antimicrobial activity of CDs-NH_2_ against all tested strains in burn wound infections. Strategies that target biofilm-forming microorganisms at wound sites may enhance infection control and promote wound healing.

## 1. Introduction

Polymicrobial infections are a leading cause of complications arising from injuries sustained by burn patients. These patients, once admitted to hospitals, face a high risk of developing hospital-acquired infections. Prolonged hospital stays further increase the likelihood of acquiring drug-resistant infections. Among the prevalent fungal and bacterial species identified in burn wound infections (BWIs) are *Candida albicans* and *Staphylococcus aureus* [1].

A significant factor contributing to the failure of burn treatment regimens and increased mortality in BWIs is biofilm formation at the wound site. Bacteria and fungi within biofilms exhibit heightened resistance to disinfectants, the host immune system, and, critically, antibiotics. These biofilms not only impair wound healing but are also thought to serve as reservoirs, facilitating the systemic spread of infection. Notably, biofilm-associated bacteria rapidly develop antibiotic resistance, with 60% of burn-related mortalities attributed to biofilm settings [2]. Biofilms provide microbial communities with significant survival advantages, demonstrating resistance to nearly all discovered antimicrobials. This resistance can increase up to 1500-fold within biofilm structures.

While numerous studies have focused on the impact of bacterial infections on the wound healing process [3], relatively little is known about the role of fungi. Fungi can cause a range of significant wound infections, from allergic reactions to life-threatening invasive fungal diseases. According to the American Burn Association's Multicenter Trials Group, 6.3% of burn patients had positive fungal cultures directly extracted from their wounds. Among the most frequently isolated pathogenic fungi are *Candida* species and filamentous fungi. Although *Aspergillus* infections in chronic wounds are rare, they deserve attention due to their potential severity, with only a few reported cases, primarily involving bone and joint ulceration [4].

The simultaneous presence of multiple species in burn wounds facilitates synergistic interactions through cell–cell adhesion and cross-feeding mechanisms. For instance, the attachment of *S. aureus* to *C. albicans* within biofilms has been well studied, providing a model for cross-kingdom interactions [5]. As a result, the development of new agents to reduce biofilm formation in burn wounds is urgently needed [6].

Given the urgent need for effective treatments against fungal and bacterial biofilms in burn wounds, novel antimicrobial agents are being explored. Promising candidates of such agents are carbon dots (CDs), a class of carbon nanoparticles characterized by small size (typically less than 10 nm) and known for their biocompatibility, low toxicity, tunable photoluminescence, high photostability, and excellent solubility in both water and organic solvents [7, 8]. Structurally, CDs consist of an amorphous or graphitic carbon *core* surrounded by a surface shell enriched with functional groups, which usually makes them highly dispersible in polar solvents. CDs can be synthesized using two main approaches, known as top-down and bottom-up. The former involves techniques such as laser ablation, arc discharge, electrochemical or chemical oxidation, and other physical or chemical fragmentation methods that break down bulk carbonaceous materials into nanoparticles. However, these strategies have been increasingly replaced by bottom-up approaches, which are mostly based on solvothermal methods, often assisted by microwave irradiation. The bottom-up approach offers better control over the structural uniformity and homogeneity of the nanoparticles while also enabling the tuning of their photoluminescent properties. Furthermore, bottom-up synthesis allows for doping CDs with nitrogen, phosphorus, and sulfur-containing molecules to enhance their electronic properties or for postfunctionalization, providing advantages that are not achievable with top-down methods. Additionally, CDs synthesized through bottom-up methods can retain the functional groups from the precursor materials on their surfaces, endowing them with a “memory shape.” Therefore, these appealing properties have made CDs a valuable tool in modern nanotechnology in a variety of fields, including chemistry, energy, and engineering, where they are used in applications as photo- and asymmetric catalysis, low-cost electronic devices for solar cells, and energy storage systems. Moreover, their unique features have made CDs an essential asset in microbiological research, with applications ranging from imaging and sensing to biomedicine, where they function as either nanocarriers or antimicrobial agents [9–11]. Notably, several studies have demonstrated that the positive surface polarization of CDs, achieved through the introduction of amine or ammonium salt groups, plays a crucial role in enhancing their antimicrobial efficacy [10].

In this study, we evaluate the activity of CDs-NH_2_ against *S. aureus*, *Escherichia coli*, *C. albicans*, and *Aspergillus brasiliensis* in both in vitro and in vivo settings. The in vivo efficacy of CDs-NH_2_ was tested using a burn model with *Galleria mellonella* larvae.

## 2. Materials and Methods

### 2.1. Synthesis, Purification, and Characterization of CDs-NH_2_

**Figure 1:**
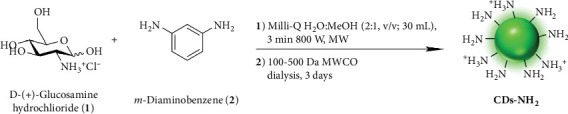
Schematic illustration of the solvothermal microwave-assisted synthesis of CDs-NH_2_.

CDs-NH_2_ were synthesized following a previously published procedure by our group (Figure 1) [12]. Specifically, 1,3-diaminobenzene (**2**) (0.55 g, 5.09 mmol) was dissolved in methanol (10 mL), sonicated for 10 min at 25°C, and subsequently added to a conical flask containing a Milli-Q water solution (20 mL) of glucosamine hydrochloride (**1**) (1.00 g, 4.64 mmol). The resulting solution was magnetically stirred for 5 min to ensure homogeneity and then irradiated at 800 W in a domestic microwave oven for 3 min. The resulting crude material was dissolved in Milli-Q water (20 mL) and centrifuged at 4000 rpm for 45 min at 25°C. The supernatant was collected and transferred into dialysis bags (MWCO = 100–500 Da) and dialyzed for 3 days against distilled water. Finally, the purified solution was filtered through syringe filters (100 nm pore size) to remove larger particles and subsequently lyophilized, yielding the final product, CDs-NH_2_, as a brown-yellowish fluffy powder. The nanoparticles were fully characterized, with detailed information provided in the supporting information.

### 2.2. Gel Electrophoresis (GE)

Freshly prepared agarose gel (1% agarose in 1× TAE buffer [40 mM Tris-acetate, 1 mM EDTA, pH 8]) was used for the analytical GE. The gels employed had dimensions of 65 (w) × 100 (l) mm (with well sizes of 5 (w) × 1.5 (l) mm). Seven microliters of stock solutions of CDs-NH_2_, **1**, and **2** (5 mg mL^−1^ in Milli-Q water) was loaded into the gel wells, and the electrophoresis was performed using the PowerPac Basic Power Supply from Bio-Rad (25 min, 120 V). The starting materials were added as control. The gel images were collected using the Gel Doc EZ Imaging, also from Bio-Rad (Epi green *λ*_exc_ = 520/545 nm).

### 2.3. Strains and Culture Conditions

The bacterial pathogens *S. aureus* subsp. *aureus* WDCM 00032 (ATCC 6538), *E. coli* WDCM 00013 (DSM 1103), and fungal pathogens *C. albicans* WDCM 00054 (ATCC 10231) and *A. brasiliensis* WDCM 00053 (DSM 1988) were used. The growth medium for *S. aureus* and *E. coli* was tryptic soy agar (TSA) and Mueller–Hinton broth (MHB). The growth media for *C. albicans* and *A. brasiliensis* were Sabouraud dextrose agar (SDA), potato dextrose agar (PDA), and RPMI-1640 medium (RPMI). Growth medium containing 50% MHB and 50% RPMI was used to grow the mixed *S. aureus* and *C. albicans* cell cultures. The culture stock was maintained in 2% glycerol, and culturing was carried out on agar plates (TSA/SDA/PDA). The pathogens were grown at 37°C. The strains and the media were obtained from Merck Life Science S.r.l. (Milan, Italy).

### 2.4. In Vitro Antimicrobial Activity of CDs-NH_2_ Against *S. aureus*, *E. coli*, *C. albicans*, and *A. brasiliensis* Strains

The antimicrobial activity was evaluated using the microdilution method, in accordance with CLSI guidelines for bacteria [13], yeasts [14], and molds [15]. *S. aureus* and *E. coli* were subcultured on TSA for 24 h at 36°C. The following day, the bacterial cultures were harvested and suspended in MHB. *C. albicans* was subcultured on SDA for 24 h at 36°C, and the inoculum was prepared the next day in RPMI. *A. brasiliensis* was subcultured on PDA for 7 days at 35°C, after which the conidia were collected, filtered, and suspended in RPMI. All inocula were transferred to 96-well plates, and CDs-NH_2_ was added at final concentrations ranging from 500 to 0.976 *μ*g/mL. The plates were incubated statically at 36°C, as specified in the protocols. After 24 or 48 h of incubation, the minimal inhibitory concentrations (MICs) were determined.

### 2.5. In Vitro Activity of CDs-NH_2_ Against Biofilm Formation

The antibiofilm activity was assessed in 48-well plates as described previously [16]. The crystal violet (CV) assay, as previously described, was employed to assess biofilm formation [17–19]. After 48 h, the cell suspensions were aspirated from the wells, and each well was washed twice with 200 *μ*L of PBS to remove nonadherent cells. The washed biofilms were air-dried for 45 min at room temperature and stained with 0.4% CV for 45 min. Following the removal of unbound CV, the wells were washed four times with PBS and then immediately destained with 95% ethanol for 45 min. A 100 *μ*L aliquot of the destained solution was transferred to a new microplate, and the absorbance was measured at 595 nm using a microplate reader. Wells containing only growth medium were used as negative controls. To minimize background interference, the absorbance values of the control wells were subtracted.

### 2.6. In Vivo Antimicrobial Activity of CDs-NH_2_ Using the *G. mellonella* Larva Model

The in vivo antimicrobial activity of CDs-NH_2_ against *S. aureus*, *E. coli*, *C. albicans*, and *A. brasiliensis* was evaluated using *G. mellonella* larvae, as described below [5, 20]. The larvae were burned using a heated steel nail with a 2 mm head embedded in cork. After inducing the burn, 200 *μ*L of CDs-NH_2_ at the concentration of 5000 *μ*g/mL was applied to the burn site. Ten minutes after CDs-NH_2_ application, 10 *μ*L of an overnight culture of the microorganism or conidia was inoculated onto the treated wound. In mixed cultures, 10 *μ*L of an overnight *S. aureus* culture was added to the wound 1 h after *C. albicans* application. Control groups included larvae with or without burns, with or without infection, and with or without CDs-NH_2_ treatment. Survival was monitored over 120 h, with larval death assessed by a color change (from light brown to dark brown) and a lack of movement when gently prodded with forceps.

### 2.7. CDs-NH_2_ Cell Uptake

The uptake of CDs-NH_2_ in *C. albicans* and *A. brasiliensis* cells was examined by epifluorescence analysis using a Leica DMRB microscope, fitted with a Leica I3 filter cube (excitation: BP 450–490 nm; emission: LP 515 nm). Micrographs were acquired using a DC500 digital camera (Leica, Wetzlar, Germany) and analyzed with Leica IM1000 image analysis software. Fungal cells were observed at 1, 5, 20, and 60 min after CDs-NH_2_ administration.

### 2.8. Statistical Analysis

Antibiofilm activity was analyzed using the one-sample *t*-test and the Wilcoxon test. Significance values are indicated as follows: *p* < 0.0001 very highly significant (⁣^∗∗∗∗^), 0.0001 ≤ *p* < 0.001 highly significant (⁣^∗∗∗^), and 0.001 ≤ *p* < 0.01 moderately significant (⁣^∗∗^). *G. mellonella* survival was displayed via Kaplan–Meier curves with a curve comparison test, *p* value: *p* < 0.0001 very highly significant (⁣^∗∗∗∗^) and 0.0001 ≤ *p* < 0.001 highly significant (⁣^∗∗∗^). Statistical data analysis was performed using GraphPad Prism 8 software (GraphPad Software Inc., La Jolla, California, United States).

## 3. Results and Discussion

**Figure 2:**
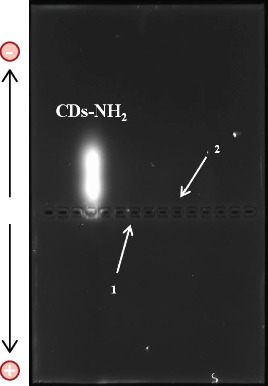
GE of CDs-NH_2_ and Precursors **1** and **2**. The stock solutions are loaded in the middle of the gel, and, after the run, the agarose gel is irradiated. Differently from the starting materials, the CDs are visualizable by a fluorescent and positive (upper—negative pole) halo.

Burn wounds represent a critical clinical challenge, as the disruption of skin integrity facilitates microbial colonization and increases the risk of severe infections. Microorganisms that infect burn wounds often form biofilms, which markedly increase their resistance to host defenses and antimicrobial treatments, making eradication extremely challenging. Inhibition of biofilm formation represents a crucial therapeutic target for preventing persistent infections. In our previous studies, we demonstrated that a hyaluronic acid–thymol conjugate and the BET inhibitor JQ1 were effective in reducing *Candida* and *Aspergillus* biofilms, respectively, two fungi that can contaminate burn wounds and complicate the healing process [6, 17]. In the present study, we investigated the activity of amino-functionalized carbon nanodots (CDs-NH_2_) against the main pathogens associated with BWIs. BWIs are commonly caused by both fungi and bacteria, including *Candida* spp., *Aspergillus* spp., *S. aureus*, and *E. coli*. Initially, Gram-positive bacteria, which are more sensitive to antibiotics, dominate in burn patients. However, over time, Gram-negative bacteria emerge, exhibiting greater resistance to treatment. Moreover, fungi such as *Candida* spp. and *Aspergillus* spp. are common in burn wounds [21], exacerbating healing due to their interaction with bacteria like *S. aureus*. The presence of *C. albicans* induces *S. aureus* biofilm formation and increases the tolerance of the bacterium to antibiotic killing [22]. To address this challenge, novel therapeutic strategies are needed to target and disrupt these biofilm-forming pathogens. To this end, our research group has synthesized CDs-NH_2_ using glucosamine and 1,3-diaminobenzene as nitrogen sources [12], which offer an innovative approach for tackling infections caused by multidrug-resistant bacteria and fungi in burn wounds. In this work, we present only a few selected characterization data of CDs-NH_2_, focusing on the most relevant for their biological activity, although some have been previously published. These nanoparticles are monodispersed with an average hydrodynamic diameter of 2.022 ± 0.064 nm and a positively charged active surface (+14.3 ± 0.21 mV), as determined by DLS, *ζ*-potential, and analytical GE. In particular, the GE analysis of the nitrogen rich CDs-NH_2_ shows clearly a bright positively charged halo in the upper (negative pole) portion of the gel. As expected, Precursors **1** and **2** are not visualizable by GE, being not fluorescent. The GE characterization confirms the synthesis of positive, reasonably because of the presence of superficial amino groups, and fluorescent nanomaterial (Figure 2).

**Figure 3:**
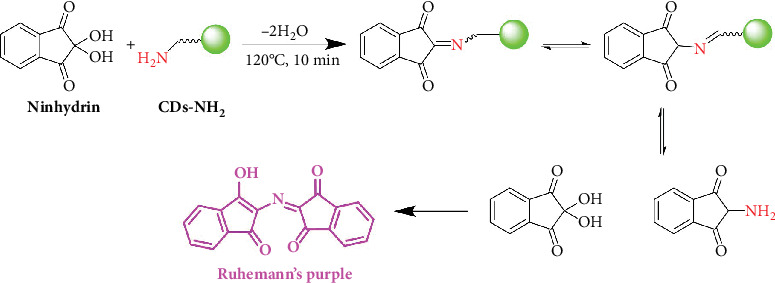
Reaction mechanism of the Kaiser test for the detection of primary amines.

Furthermore, in this work, we focused on the content of active nitrogen, which is crucial for biological activity. To this end, the primary amine content on the surface of CDs-NH_2_ was quantified using the Kaiser test, a spectrophotometric assay specifically designed for the determination of primary amines, following a literature procedure [23]. This method exploits the characteristic absorption peak centered at 560–570 nm, which corresponds to the formation of a conjugated chromophore between ninhydrin and primary amine groups, commonly referred to as Ruhemann's purple (Figure 3). This method is employed for the detection of primary amines containing at least one proton on the C_*α*_ position, operating through a multistep mechanism under basic conditions. However, the test tends to underestimate the actual content of primary amino groups, as Ruhemann's purple suffers from instability under these conditions, and some amino groups may remain unreacted due to significant steric hindrance [24]. The analysis was repeated a minimum of four times for three different batches of CDs-NH_2_ synthesized on separate occasions. The mean value of primary amines per milligram of CDs-NH_2_ was found to be 0.931 ± 0.143 *μ*mol/mg (Figure S1), yielding reproducible data and confirming the high density of amino groups on the nanoparticles' surface. It is reasonable to assume that these amino groups are likely responsible for the nanoparticles' antimicrobial properties, as well as for their promising antioxidant activity, which we have recently reported [12].

The presence of nitrogen was also confirmed through XPS analysis (supporting information), with peaks centered at 400 and 402 eV corresponding to amino and ammonium nitrogen. Additional confirmation was provided by ATR-FTIR spectroscopy and elemental analysis, as detailed in our previous work [12]. Moreover, the ^1^H-NMR spectrum of CDs-NH_2_ further confirmed the presence of an aromatic *core* and a nitrogen-functionalized shell (Figure S2).

The absorption and emission spectra were recorded to evaluate their potential for applications in bioimaging: Specifically, CDs-NH_2_ exhibit a strong absorption peak centered at 218 nm, attributed to the electronic transitions *π*-*π*∗ of the aromatic *core*, along with lower intensity peaks at 363 and 300 nm, ascribed to the n-*π*∗ transitions of lower energy. On the other hand, CDs-NH_2_ display bright green fluorescence, nearly independent of excitation, with the emission peak centered at 542 nm and the maximum excitation at 460 nm (Figure S3).

This study assessed the activity of CDs-NH_2_ against *S. aureus*, *E. coli*, *C. albicans*, and *A. brasiliensis* both in vitro, on planktonic cells and biofilms, and in vivo, using a BWI model. We previously investigated the antifungal activity of CDs featuring different surface charges. Our study revealed that positively charged CDs—specifically, the CDs-NH_2_ variant—effectively inhibited *Candida* biofilm formation and adhesion, likely through interactions with the fungal cell surface [12]. In the present study, the activity of CDs-NH_2_ was also assessed against a mixed biofilm of *S. aureus* and *C. albicans*, in vitro and in vivo within the same BWI model.

**Figure 4:**
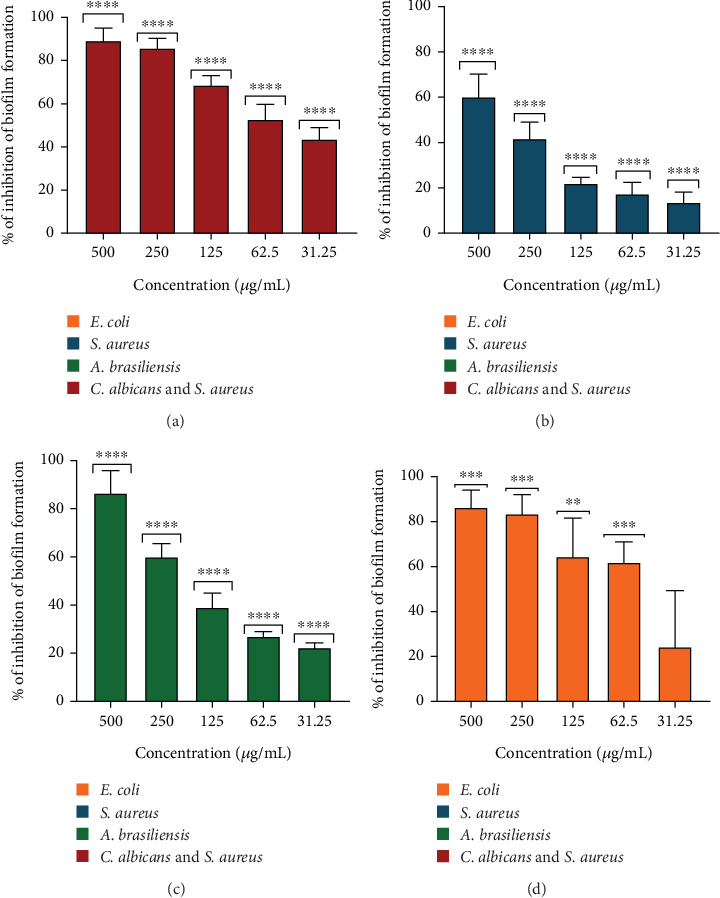
Inhibition of (a) *Escherichia coli*, (b) *Staphylococcus aureus*, (c) *Aspergillus brasiliensis*, and (d) *Candida albicans*–*S. aureus* coculture biofilm formation in response to CDs-NH_2_. Data were analyzed using the one-sample *t*-test and the Wilcoxon test. Significance levels: ⁣^∗∗∗∗^*p* < 0.0001,^∗∗∗^0.0001 ≤ *p* < 0.001, and ⁣^∗∗^0.001 ≤ *p* < 0.01.

CDs-NH_2_ inhibited *S. aureus*, *E. coli*, *C. albicans*, and *A. brasiliensis* planktonic cell growth with GMMIC values of 210, 500, 397, and more than 500 *μ*g/mL, respectively. CDs-NH_2_ were used at a concentration ranging from 500 to 31.25 *μ*g/mL to evaluate the inhibition of biofilm formation. They exhibited the capacity to inhibit biofilm formation in a dose-dependent manner (Figure 4). CDs-NH_2_ demonstrated significant activity in reducing *E. coli* biofilm formation by 85.72% at concentrations of 250 *μ*g/mL (Figure 4a). The *S. aureus* biofilm formation was inhibited by 60% at the concentration of 500 *μ*g/mL (Figure 4b). *A. brasiliensis* biofilm formation was inhibited by 86.57% at a concentration of 500 *μ*g/mL and 60% at a concentration of 250 *μ*g/mL (Figure 4c). Our previous studies demonstrated, and additional observations (not included here) further confirmed, that CDs-NH_2_ inhibit *C. albicans* biofilm formation by approximately 95% at a concentration of 500 *μ*g/mL. The inhibition of mixed biofilm formation of *S. aureus* and *C. albicans* was 83.60% at concentrations of 250 *μ*g/mL. An inhibition of 64.39% was detected following treatment with 125 *μ*g/mL CDs-NH_2_ (Figure 4d). CDs-NH_2_ showed activity against *S. aureus*, *E. coli*, *C. albicans*, and *A. brasiliensis*, all relevant pathogens in BWIs. They were effective against both single-species and mixed *S. aureus–C. albicans* biofilms, a polymicrobial association known to increase antibiotic tolerance. Their inhibitory effect may involve a dual mechanism, not only limiting microbial proliferation but also interfering with early adhesion processes required for biofilm initiation.

**Figure 5:**
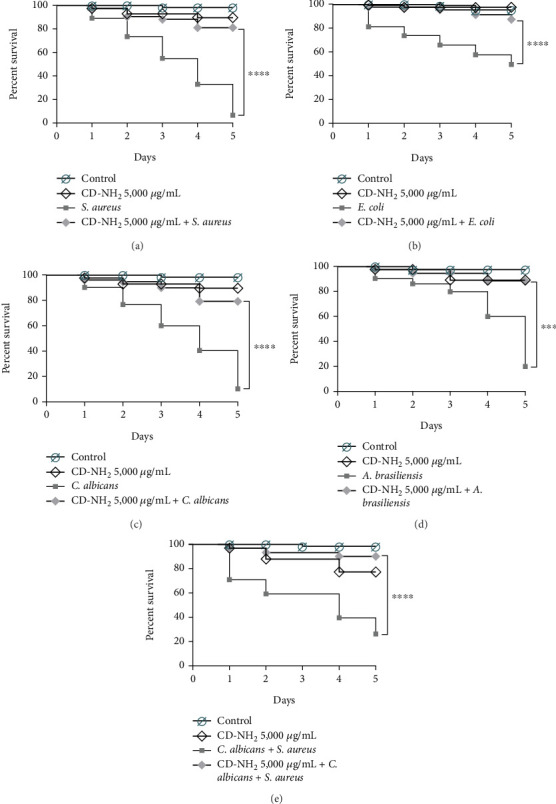
*G. mellonella* survival rate after (a) *Staphylococcus aureus*, (b) *Escherichia coli*, (c) *Candida albicans*, (d) *Aspergillus brasiliensis*, and (e) *C. albicans*–*S. aureus* coculture infection and treatment with 5000 *μ*g/mL of CDs-NH_2_. Survival of *Galleria mellonella* was assessed by Kaplan–Meier analysis with curve comparison; significance: ⁣^∗∗∗∗^*p* < 0.0001 and ⁣^∗∗∗^0.0001 ≤ *p* < 0.001.

Furthermore, in vivo tests were conducted. The complexity of burn wound trauma and infection necessitates the use of an in vivo model for developing novel therapeutics. While multiple in vivo models are available, the murine model is the most commonly used. However, mammalian BWI models pose logistical challenges, are not well-suited for high-throughput screening, and raise significant ethical and animal welfare concerns. The use of *G. mellonella* larvae has become increasingly common in recent decades as a model system for studying bacterial and fungal infections and for evaluating the effectiveness of new antimicrobial drugs. This approach is employed as a preliminary step before progressing to preclinical trials involving mammals, thereby emphasizing the commitment to the principles of the 3Rs (replacement, reduction, and refinement) in animal research. Recently, the *G. mellonella* model for burn wound and infection has been established [13]. This model offers several advantages over traditional animal models, including lower costs, easier maintenance, and reduced ethical concerns. Furthermore, in vivo tests conducted with *G. mellonella* larvae demonstrated a high percentage of larval survival when infected with microorganisms after treatment with CDs-NH_2_ (Figure 5). Larvae infected with *S. aureus* (Figure 5), *E. coli* (Figure 5), *C. albicans* (Figure 5), and *A. brasiliensis* (Figure 5) and treated with CDs-NH_2_ showed a significant decrease in mortality, with survival increasing to 81.18%, 87.20%, 79.10%, and 88.26%, respectively, compared to non-CDs-NH_2_-treated larvae. Larvae with induced burns, treated with CDs-NH_2_ and coinfected with *C. albicans* and *S. aureus*, showed a 90.13% reduction in mortality (Figure 5). In vivo tests using the *G. mellonella* burn wound model confirmed that CDs-NH_2_ markedly reduced mortality from bacterial and fungal infections, in both single-species and mixed settings. Survival exceeded 80% in all treated groups, including the particularly challenging *C. albicans*–*S. aureus* coinfection, underscoring the potential of these nanomaterials in managing complex wound-associated infections. The protective effect observed may be explained by a dual mechanism, in which CDs-NH_2_ not only inhibit microbial proliferation but also, hypothetically, interfere with microbial colonization and the early steps of biofilm formation in burn wounds.

**Figure 6:**
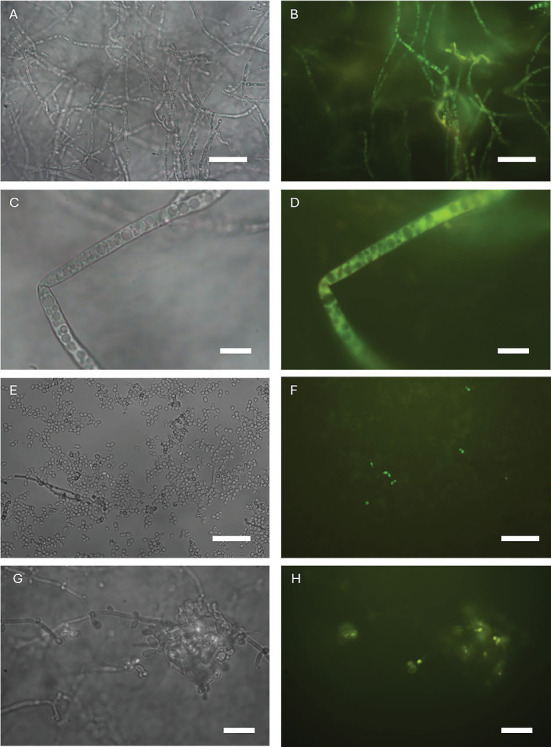
Analysis of CDs-NH_2_ cell uptake by comparing micrographs of (A–D) *Aspergillus brasiliensis* hyphae and (E–H) *Candida albicans* hyphae and conidia under (A, C, E, G) bright field or (B, D, F, H) fluorescence. Scale bars (A, B, E–H) 50 and (C, D) 10 *μ*m.

Some authors have reported that the antimicrobial mechanisms of CDs against bacteria are due to penetration facilitated by electrostatic interactions, disruption of the cell structure leading to leakage of intracellular components, and induction of oxidative stress along with the inhibition of antioxidant enzymes [19]. The intrinsic fluorescence of CDs-NH_2_ was exploited to verify their ability to penetrate fungal cells. Observations were made on in vitro cultured *C. albicans* and *A. brasiliensis* treated with CDs-NH_2_. The observations on untreated cells showed negligible autofluorescence of the fungal cells. Analysis of cells treated with CDs-NH_2_ showed a fluorescent signal within the fungal cells of both species as early as 5–10 min after administration (Figure 6). The fluorescence signal inside the cells was much more intense than that observed in the background, suggesting that the concentration of CDs-NH_2_ was much higher inside the cells than in the external environment. Within the cells, the fluorescence signal was intense in the cytoplasm and weak in the vacuoles, indicating compartment-specific accumulation [5]. The promising activity of CDs-NH_2_ may be attributed to the presence of a positively charged surface polarization due to the presence of aminic functional groups, resulting in a prominent interaction with the fungal membrane leading to internalization. We view this study as an initial step toward thoroughly exploring the antimicrobial capabilities and potential applications of CDs-NH_2_. However, further research is necessary to fully investigate the potential of CDs-NH_2_ as an antimicrobial agent for treating infections caused by *S. aureus*, *E. coli*, *C. albicans*, and *A. brasiliensis* in burn wounds.

## Data Availability

The data that support the findings of this study are available from the corresponding author upon reasonable request.

## Supporting Information

Supporting Information Additional supporting information can be found online in the Supporting Information section. The supporting information includes the following: determination and quantification of primary amino groups on CDs-NH_2_ (Equation S1 and Figure S1), XPS analysis (Table S1), ^1^H-NMR characterization (Figure S2), and optical properties of CDs-NH_2_ (Figure S3).
