# Supplementary material for: Amino-Functionalized Carbon Nanodots Inhibit Biofilms and Infections in a Burn Wound Model, Both Caused by Staphylococcus aureus, Escherichia coli, Candida albicans, and Aspergillus brasiliensis
Source: Int J Microbiol. 2025 Oct 14;2025:8824725. doi: 10.1155/ijm/8824725 (PMC12539990; doi:10.1155/ijm/8824725)
Supplement: Supporting Information — Additional supporting information can be found online in the Supporting Information section. The supporting information includes the following: determination and quantification of primary amino groups on CDs-NH2 (Equation S1 and Figure S1), XPS analysis (Table S1), 1H-NMR characterization (Figure S2), and optical properties of CDs-NH2 (Figure S3). [file 8824725.f1.docx]

**Supplementary Material**

Kaiser Test of CDs-NH_2_

Kaiser Test was employed to assess the content of primary amino groups on the surface of CDs-NH_2_, using a commercially available kit from Merck. Briefly, approximately 1 mg of different batches of CDs-NH_2_ (referred to as S1, S2 and S3) were dissolved in 250 µL of water in a vial. Suddenly, 75 μL of a phenolic solution in 80% ethanol, 100 μL of a KCN solution in pyridine/water, and 75 μL of a ninhydrin solution in 6% ethanol were added. The vial was placed into a preheated silicone oil bath at 120°C and allowed to react for 10 minutes. Afterward, the reaction was quenched and diluted with a 1:1 solution of water and ethanol to achieve a final volume of 6 mL. Then, 500 µL of this solution was transferred into a polystyrene cuvette, diluted with 2 mL of the diluting solution and the UV absorption spectrum was acquired. A blank solution not containing CDs-NH_2_ was also reacted and used as a reference. The absorbance value at 560 nm was recorded and used to estimate the content of primary amino groups on the carbon dots surface according to **Equation S1**:

$$\frac{{\mu mol}_{{NH}_{2}}}{{mg}_{CDs}}= \frac{Abs \left( 560 nm \right)\cdot{10}^{3}}{\varepsilon(M^{-1}\cdot{cm}^{-1})\cdot l (cm)\cdot c (mg/mL)}$$

Equation S1: Evaluation of primary amines in CDs-NH_2_ through the Kaiser Test.

where $\varepsilon$= 15000 M^-1^ ‧ cm^-1^ for Ruhemann’s purple, $l$ = 1 cm and $c$ represents the mass concentration in mg/mL. For the three samples, at least for measures were performed, and the mean value across all experiments was reported as 0.931 ± 0.143 µmol/mg.


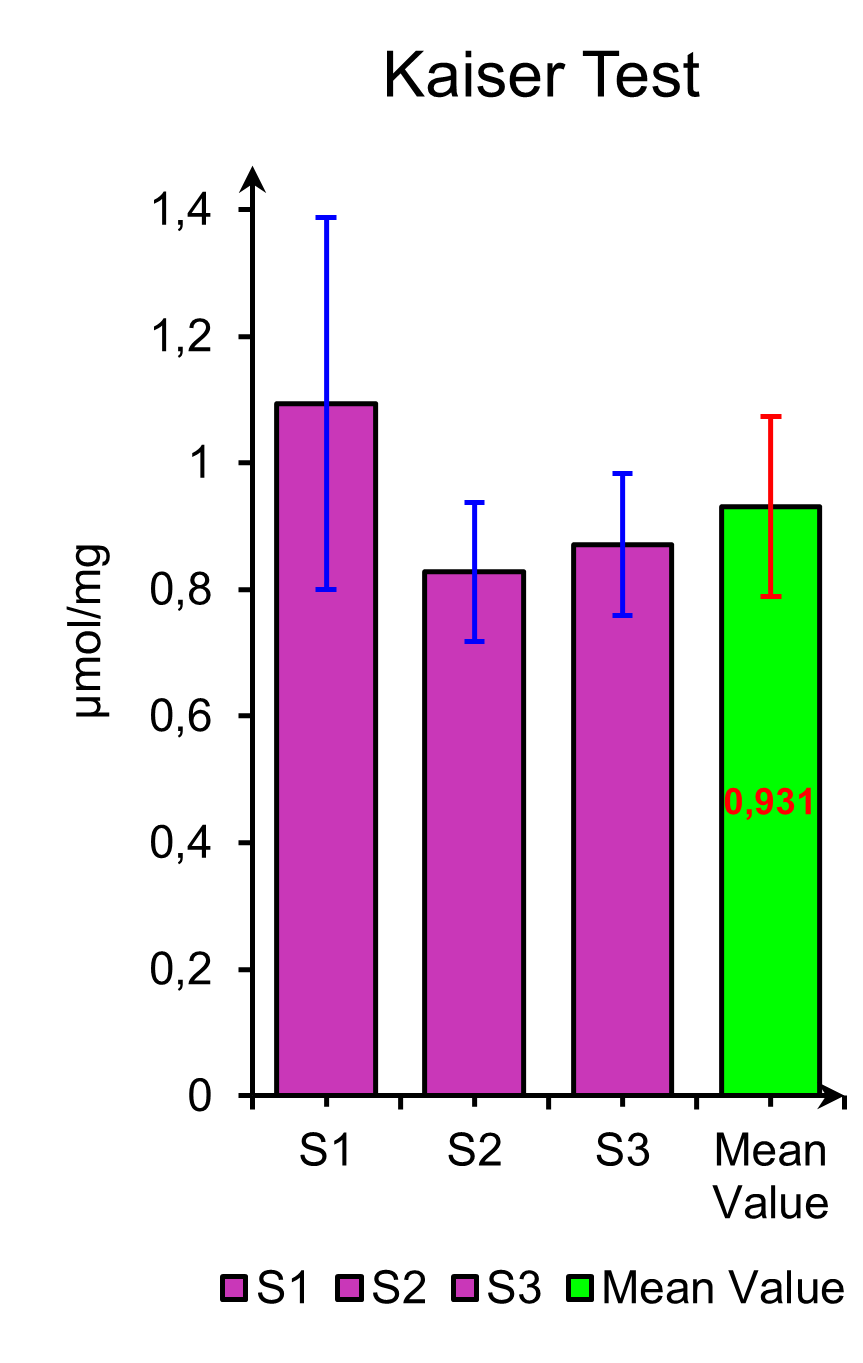


Figure S1: Kaiser Test results for the determination of primary amines in different batches of CDs-NH₂ (S1, S2, S3). Welch’s t-test analysis confirmed that the differences between groups (S1, S2, and S3) are not statistically significant (p > 0.05).

XPS analysis

Table S1 presents the main XPS peaks corresponding to the heteroatoms present in the carbon dots, highlighting key surface chemical features.

| Element | Binding Energy (eV) | Functional Group |
| --- | --- | --- |
| N | 400, 402 | Amino, ammonium |
| O | 533 | Hydroxyl |
| Cl | 198, 200 | Chloride |

Table S1: XPS functional group assignments for CDs-NH_2_: key binding energy peaks corresponding to heteroatom functionalities.

^1^H-NMR characterization

The ^1^H-NMR spectrum (**Figure S2**) was recorded at room temperature by dissolving CDs-NH_2_ in D_2_O at a concentration of approximately 20 mg/mL. The analysis was performed on a 400 MHz Bruker Avance III spectrometer and spectrum was processed by using MestReNova 12.0.0-20080 (Mestrelab research S.L.). Residual internal solvent was used as reference.


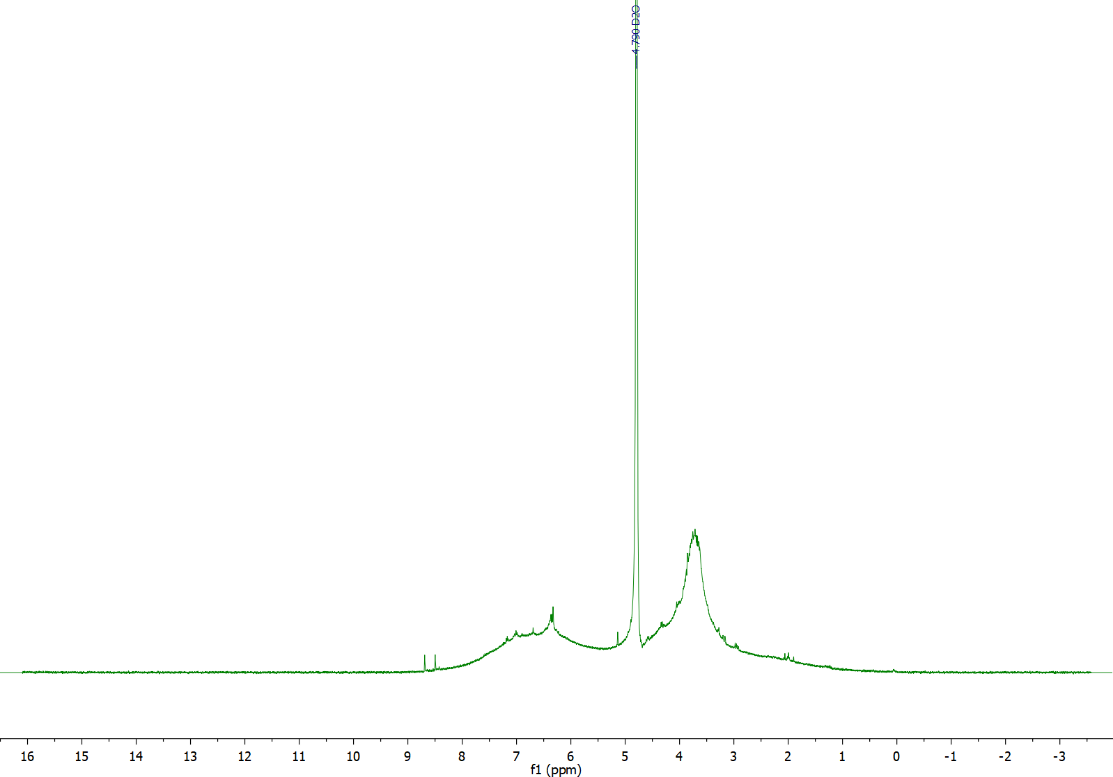


Figure S2: ^1^H-NMR spectrum of CDs-NH_2_ in D_2_O.

Optical properties

The UV-Visible and fluorescence spectra, shown on the left (green) and right sides of the image, were recorded at room temperature by dissolving CDs-NH_2_ in Milli-Q water at a concentration that ensures absorbance lower than 0.1, minimizing autofluorescence effects. The data were analyzed using OriginPro 2018.





Figure S3: Absorption (green, left) and emission spectra (right) of CDs-NH_2_. The emission spectra were recorded by exciting CDs-NH_2_ in the range of 360 to 560 nm, with measurements taken every 20 nm.
